# The Crosstalk Between NETs and the Complement Cascade: An Overview in Nephrological Autoimmune Disease

**DOI:** 10.3390/ijms26062789

**Published:** 2025-03-20

**Authors:** Xhuliana Kajana, Gianluca Caridi, Maurizio Bruschi, Sonia Spinelli, Francesca Lugani, Gian Marco Ghiggeri, Edoardo La Porta, Gabriele Mortari, Enrico E. Verrina, Andrea Angeletti, Carolina Bigatti

**Affiliations:** Nephrology, Dialysis and Transplantation Unit, IRCCS Istituto Giannina Gaslini, 16145 Genoa, Italy; xhulianakajana@gaslini.org (X.K.); gianlucacaridi@gaslini.org (G.C.); mauriziobruschi@yahoo.it (M.B.); soniaspinelli@gaslini.org (S.S.); francescalugani@gaslini.org (F.L.); gmarcoghiggeri@gaslini.org (G.M.G.); edoardolaporta@gaslini.org (E.L.P.); gabrielemortari@gaslini.org (G.M.); enricoverrina@gaslini.org (E.E.V.); carolina.bigatti.cb@gmail.com (C.B.)

**Keywords:** complement cascade, Neutrophil Extracellular Traps, autoimmunity, Systemic Lupus Erythematosus, ANCA vasculitis, antiphospholipid syndrome, eculizumab, avacopan

## Abstract

The complement cascade and Neutrophil Extracellular Traps (NETs) represent fundamental tools in protecting the host from foreign pathogens. Complement components and relative fragments, classically assigned to the innate immunity, represent a key link with the humoral immune response. NETs are a crucial component of the innate immune response, consisting of chromatin release from activated neutrophils. These web-like structures facilitate pathogen entrapment and elimination through proteolytic degradation and antimicrobial effectors. Previous findings suggested complement components and NETs have a significant role in the pathogenesis of several diseases characterized by inflammation, such as autoimmune and infectious diseases. However, the crosstalk between NETs and the complement cascade has only recently been investigated, and several aspects still need to be fully clarified. Recent evidence seems to suggest a bidirectional link between the complement cascade and NETosis. We here present the interaction between complement components and NETs in specific autoimmune diseases that mostly affect the kidney, such as systemic lupus erythematosus, Antineutrophilic Cytoplasmic Antibody (ANCA)-associated vasculitis and antiphospholipid syndrome. The mechanisms reported here may represent specific targets for the development of possible therapeutic strategies.

## 1. Introduction

The complement cascade plays a fundamental role in protecting the host from foreign pathogens, serving as a functional bridge between the adaptive and humoral immune responses. The complement cascade can be activated through three distinct pathways: the classical (CP), lectin (LP) and alternative (AP) pathways. Each pathway converges on C3 convertases, which are enzymatic multimeric protein complexes, resulting in the cleavage of inactive C3 protein into the functional fragments C3a and C3b. The latter triggers formation of C5 convertase and cleavage of C5, resulting in formation of the membrane attack complex (MAC, C5b-9) and subsequent pathogen lysis. Along with MAC, other soluble and surface-bound split products of the complement system cooperate in the inflammatory response. C3b may also cover foreign pathogens, resulting in opsonization, through complement receptor 1 (CR1) on neutrophils and other phagocytes, facilitating the ingestion and degradation of pathogens [1,2].

In 2004, Brinkmann et al. [3,4] reported NETosis as a novel way of entrapping and killing pathogens. NETosis represent a regulated form of neutrophil cell death that contributes to the host defense through the formation of neutrophil extracellular traps (NETs). Neutrophils are activated and induced to release NETs, composed of chromatin together with antibacterial peptides and enzymes, through the interaction of microbial products and immune complexes [5].

**Figure 1:**
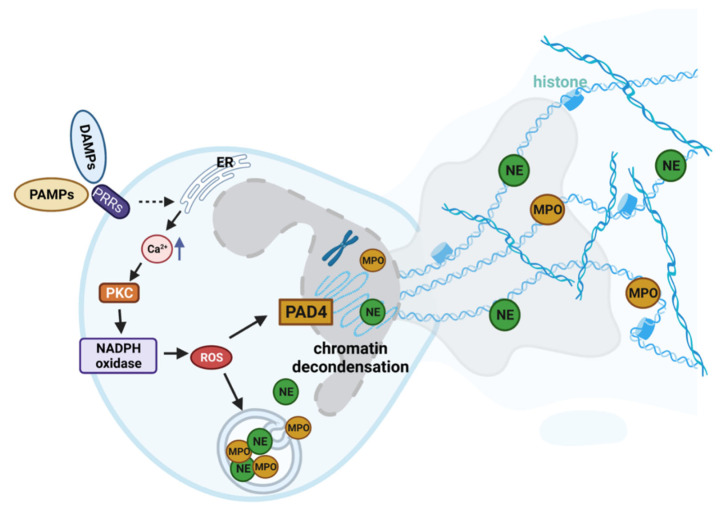
**Representative inflammatory mechanisms leading to NETosis.** Neutrophils are activated by damage-associated molecular patterns (DAMPs) and pathogen-associated molecular patterns (PAMPs) through binding with Toll-like receptor (TLR) or IgG-Fc receptors, with consequent release of Ca^2+^ by the ER. The cytoplasmic increase of Ca^2+^ activates the protein Kinase-C (PKC) through the RAF–MEK–MAPK/ERK pathway, a critical intracellular signaling pathway that regulates cell proliferation, differentiation, survival, and apoptosis. Ca^2+^ influx and PKC activation have pivotal roles in stimulating NADPH oxidase activity on the cell surface, promoting the production of reactive oxygen species (ROSs). The increase in ROSs induces the release of enzymes such as neutrophil elastase (NE) and myeloperoxidase (MPO). These, together with peptidyl arginine deiminase 4 (PAD4) are commonly localized in the nucleus and involved in chromatin decondensation. The NET produced from neutrophils is therefore composed of DNA, histones, other nuclear components, and intracellular enzymes such as NE and MPO.

In the extrusion of NETs, the activation of oxidative enzymes is fundamental. Among other things, NADPH induces the release of neutrophil elastase (NE) and myeloperoxidase (MPO) from cytoplasmic granules. NE induces histone degradation leading to DNA decondensation. When the nuclear membrane disintegrates, the decondensed chromatin mixes with cytosolic and granular proteins, forming NETs, which are released outside the cell (Figure 1). Circulating NETs are then degraded by plasma DNases and subsequently removed by macrophages [6].

Recent evidence suggested a bidirectional link between complement cascade and NETosis, which will be the topic of this review article [7]. We will also discuss recent findings on interactions between the complement cascade and NETosis in different clinical conditions, such as autoimmune disease and infectious diseases.

Herein, we here explore the complex bidirectional interplay between the complement cascade and NETosis, with a focus on autoimmune diseases with renal involvement. We highlight recent breakthroughs in understanding how complement activation can drive NET formation, and conversely, how NETs can amplify complement-mediated inflammation and tissue damage. Additionally, we provide a concise yet thorough overview of emerging therapeutic strategies designed to target NETs and complement components, with the goal of mitigating immune dysregulation and organ damage in these disorders.

## 2. Interplay of NETosis and Complement Cascade

In the last decade, several findings supported the deep interplay between components of the complement cascade and NETosis. Preliminary data reported that neutrophils from C3^−/−^ mice or from C3a receptor (C3aR)-deficient mice did not form NETs [7]. In line with this, when C3 knock-out mice were given exogenous C3-containing serum from wild-type mice, the NETotic ability of neutrophils was restored, enforcing the importance of the complement system for NETosis [7].

### 2.1. Complement Cascade Activates NETosis

Once activated, neutrophils release complement factors of the AP (Factor B and properdin) against invading pathogens. The released properdin, through an autocrine mechanism, deposits on the neutrophil membrane, leading to the cleavage of C3 and complement activation. The complement cascade activation on the neutrophil membrane leads to lysing of the neutrophils with consequent NETosis. On the neutrophil membrane, complement activation is balanced and counteracted by Factor H, an inhibitor of the AP [8,9].

Palmer et al. [10] recently investigated the DNA release induced by several bacteria such as Staphylococcus aureus and other oral pathogens. The adding of serum to bacteria culture markedly enhanced NET release. In accordance, NETosis may be prevented by inactivation of the serum complement or by inhibition of bacterial complement receptor type 1. These mechanisms suggest that the ability of a pathogen to induce NETosis might inversely correlate with its ability to evade the complement system and opsonization [11,12,13].

### 2.2. NETs Activate Complement Cascade

Since the 1970s, studies have demonstrated that neutrophils and the complement cascade act synergistically in counteracting pathogens. Several reports showed that proteins purified from azurophilic granules of neutrophils were able to cleave C3 and C5, such as MPO, which cleaves C5 into active fragments [14,15]. Similarly, cathepsin G and neutrophil elastase can activate the complement system through the cleavage of C3 [16].

**Figure 2:**
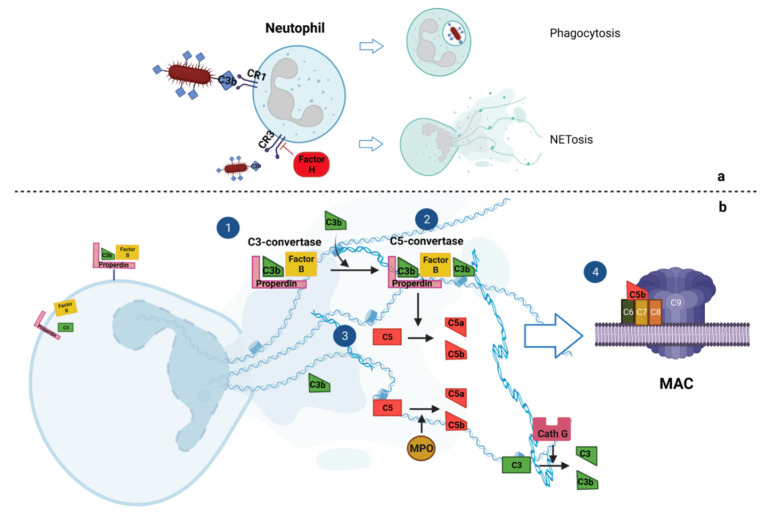
**Main mechanisms of interplay between complement cascade and neutrophil extracellular traps (NETs).** (**a**) The binding between complement receptors 1 (CR1) and 3 (CR3) on neutrophils with a C3b-opsoninized pathogen commonly leads to phagocytosis and NETosis. Factor H, on neutrophil membranes, limits the inflammatory response, acting as an inhibitor of the link between C3b and CR3. Neutrophils commonly express Factor B and Properdin, which are fundamental for the formation and stabilization of C3-convertase, with consequent activation of the complement cascade. (**b**) This occurs both at the membrane level and on the NETs. C3b binding to stabilized C3-convertase (1) leads to the formation of C5-convertase (2), which converts C5 into C5a and C5b (3). C5b with C6, C7 and C8 forms the membrane attack complex (MAC) complex (4). Myeloperoxidase (MPO) and cathepsin G, in NETs, can respectively cleave C5 and C3.

More recent studies supported the hypothesis that NETs can activate the complement system to alarm the immune system. Complement activation occurs not only on the neutrophil membrane, as reported above, but also on released NETs. Properdin, Factor B and C3 have been found to be deposited on NETs [17,18,19]. MPO, cathepsin G and proteinase 3, which are largely expressed in neutrophils and are incorporated into NETs, can activate the complement cascade after NETosis through binding with circulating properdin and with properdin on NETs [13]. Correspondingly, when isolated NETs were incubated with non-inactivated serum, complement components were consumed as a result and the assembled MAC complex was found to be deposited on NETs [9,19]. In all cases, complement activation by NETs is strongly reduced when DNase-I is added to disrupt the NET structures [9]. Similarly to what happens on neutrophils, Factor H is also recruited onto NETs to counteract and to balance the C3b activation [20] (Figure 2).

Which complement pathway is mainly activated by NETs is still largely debated. However, in vitro studies suggested that the complement cascade in NETs may be mainly activated through the AP [17,19].

## 3. NETosis and Complement Components in Renal Autoimmune Disease

Systemic autoimmune diseases are characterized by the failure of the immune system to differentiate self from non-self. These conditions are associated with significant morbidity and mortality, and they can affect many organs and systems, with significant clinical heterogeneity [21,22].

Systemic autoimmune diseases, such as systemic lupus erythematosus (SLE), ANCA-associated vasculitis (AAV) and antiphospholipid antibody syndrome (APS), present different clinical manifestations but share significant pathogenic overlaps. These diseases were classically related to dysfunctions of humoral immunity, but studies over the last two decades also pointed out the fundamental role of innate immunity and the type I interferon pathway [23,24,25].

Despite their beneficial role in host defense, NETs contribute to the pathogenesis of various autoimmune diseases. The balance between NET release and removal is crucial, as impaired clearance can exacerbate immune responses against DNA, histones, and intracellular proteins, including citrullinated peptides, myeloperoxidase, and proteinase 3 associated with NETs [26].

### 3.1. Systemic Lupus Erythematosus

SLE represents the archetypal systemic autoimmune disorder, characterized by diverse clinical manifestations affecting the kidneys, skin, synovial joints, lungs, heart, brain, and blood vessels. Renal involvement occurs in a substantial percentage of SLE patients, ranging from 28% to 74%, and is associated with increased mortality rates [27]. Despite therapeutic interventions, a noteworthy proportion of SLE patients progress to end-stage kidney disease (ESKD), and the disease’s impact is exacerbated by the risk of recurrence following kidney transplantation [27,28,29].

The pathogenesis of SLE is multifactorial, marked by a breakdown in immune tolerance against self-antigens, leading to the production of autoantibodies. The intricate mechanisms underlying SLE pathogenesis remain incompletely understood, with a pivotal role attributed to defective clearance of apoptotic cell debris, exposing nuclear antigens. This initial event triggers an innate inflammatory response mediated by toll-like receptors (TLR), subsequently amplifying T cell and B cell responses against autoantigens [30].

Earlier suggestions implicated neutrophil dysfunction in SLE patients, characterized by delayed clearance, heightened apoptosis, and oxidative metabolism [31,32,33]. Notably, individuals with more severe clinical manifestations exhibit increased expression of neutrophil-specific genes [32].

Research has shown that neutrophils from healthy individuals exhibit increased NET formation when exposed to sera from SLE patients compared to sera from healthy controls [34]. Similarly, SLE patient neutrophils have a higher propensity for NET release, with both intrinsic and extrinsic factors contributing to their activation and pathogenic role in SLE [35].

A unique subset of neutrophils, known as low-density granulocytes, is more abundant in SLE and releases NETs more promptly [36]. SLE patients also show an impaired ability to degrade NETs via DNase activity, a critical mechanism that regulates extracellular DNA levels [37,38].

SLE is also characterized by circulating autoantibodies that target NET components, forming immune complexes upon binding with DNA or RNA. These complexes, upon tissue deposition, can activate plasmacytoid dendritic cells, which in turn trigger TLR7 and TLR9 pathways. This response elevates type I interferon levels, promoting immune cell activation and further NET formation [39]. Additionally, C1q may interact with antibodies against NET components, potentially reducing DNase activity, while the generation of anti-DNase antibodies has been noted in some cases [40]. Consequently, impaired NET clearance is a key pathogenic mechanism in SLE, associated with lower complement levels (C3, C4) and higher susceptibility to glomerulonephritis in patients with elevated NET levels [41,42].

The deep interplay between NETs and complement components in SLE is further substantiated by mutations in DNase genes, characteristic of monogenic forms of SLE [43,44]. In 2001, Yasutomo et al. [45] delineated two cases with mutations in exon 2 of DNASE1, presenting symptoms and laboratory findings compatible with SLE, exacerbated by renal involvement with proliferative glomerulonephritis and IgG and C3 glomerular deposition. Similar findings have been documented in subsequent studies [46,47]. Despite clinical heterogeneity, all these cases exhibited reduced circulating complement components and an augmented type I interferon signature, both hallmark features of SLE [47].

### 3.2. Anti-Neutrophil Cytoplasmic Antibody-Associated Vasculitis

Vasculitis encompasses a spectrum of autoimmune disorders characterized by leukocyte infiltration and activation within the vascular endothelium, leading to inflammation and tissue damage. AAV are defined by the presence of pathogenic autoantibodies targeting neutrophil-derived antigens, such as myeloperoxidase and proteinase 3 [48]. Renal involvement is a hallmark of AAV, with pauci-immune glomerulonephritis being the predominant manifestation [2]. Despite advancements in therapeutic strategies, the risk of progressive kidney failure remains substantial in AAV patients [49,50]. AAV is characterized by excessive neutrophil activation, leading to increased formation of NETs. NETs function as pro-inflammatory scaffolds, promoting endothelial injury, complement activation, and autoantibody production [51]. NET-driven complement activation plays a pivotal role in renal vasculitis, exacerbating glomerulonephritis through C5a-dependent neutrophil recruitment and inflammation. Notably, patients with AAV exhibit elevated circulating NET levels, and in vitro studies demonstrate that neutrophils exposed to serum from AAV patients display an increased propensity for NETosis, with NETs exhibiting prolonged persistence in circulation [51].

NETs have been shown to directly activate the alternative complement pathway, thereby amplifying inflammation and endothelial damage in AAV [17]. The C5a–C5aR1 axis plays a central role in this pathogenic cycle, as C5a promotes NET formation, establishing a self-perpetuating loop of neutrophil activation and complement amplification [52]. In addition, C5b-9 (membrane attack complex, MAC) deposition on endothelial cells further exacerbates neutrophil-mediated vascular injury. NETs isolated from bronchoalveolar lavage fluid and renal biopsies of patients with active AAV display elevated levels of neutrophil-derived tissue factor, which contributes to thrombosis and inflammation [53]. Furthermore, Nakazawa et al. [31] identified a complex interplay between NETs, macrophages, and Toll-like receptor 9-mediated citrullination, which further amplifies the inflammatory cascade in AAV [31,54].

A reduction in circulating DNase I activity is another key contributor to NET persistence and complement activation in AAV, leading to impaired NET clearance [55]. This excessive NET burden has two major consequences: sustained complement activation, which perpetuates endothelial damage, and autoantibody generation, as NETs serve as scaffolds for ANCA production. Indeed, NETs have been identified as potential autoantigen-presenting structures, driving ANCA production against MPO and PR3, thereby exacerbating tissue inflammation and kidney injury [51].

The interplay between NETs and complement activation is fundamental to the progression of renal vasculitis in AAV. This interaction contributes to persistent inflammation and endothelial injury, primarily via C5a-mediated neutrophil recruitment and activation. Furthermore, it fosters thrombosis and tissue damage, driven by NET-bound tissue factor and complement deposition, and autoantibody generation, with NETs acting as antigen-presenting platforms for ANCA production. Given the central role of NETs and complement in AAV pathogenesis, targeting both pathways simultaneously represents a promising therapeutic approach, potentially mitigating inflammatory and thrombotic complications while preserving renal function.

### 3.3. Antiphospholipid Syndrome

In addition to SLE and AAV, NETs have also been implicated in other autoimmune renal diseases, including APS. APS is an autoimmune disorder characterized by the presence of antiphospholipid antibodies, which predispose individuals to arterial and venous thrombosis, primarily affecting the pulmonary and renal vasculature [56].

Patients with APS exhibit higher serum levels of NETs compared to healthy controls. Additionally, antiphospholipid antibodies purified from APS patients directly induce NETosis in control neutrophils, supporting a role for NETs in enhancing thrombin generation and contributing to the prothrombotic state of APS [42].

Beyond their contribution to thrombosis, recent evidence suggests a critical interplay between NETs and complement activation in APS, which remains only partially elucidated. Antiphospholipid antibodies trigger classical complement activation via C1q binding, amplifying the inflammatory and prothrombotic response [57]. In parallel, NET components, as previously described, can bind C1q, further potentiating complement activation and driving endothelial dysfunction [57]. Conversely, C5a enhances NET formation, creating a self-reinforcing cycle of immune activation and thrombosis [58].

Moreover, C5b-9 deposition on endothelial cells contributes to neutrophil activation and further NET release, exacerbating vascular damage. Elevated circulating NET remnants in APS patients have been correlated with disease severity and thrombotic burden, suggesting their potential role as a biomarker for APS-related vascular injury [59].

Therefore, similarly to other autoimmune renal diseases, targeting both NETs and complement components could offer novel strategies for APS management. Overall, while the role of NETs in APS pathogenesis is well-established, their interplay with complement components remains an active area of investigation. Further studies are needed to define the mechanistic links between NETosis, complement activation, and thrombogenesis in APS, potentially unveiling new targeted therapeutic approaches for high-risk or refractory APS cases.

## 4. Possible Treatments

Understanding the pathological mechanisms linking complement components, NETs, and renal involvement in autoimmune disease is fundamental for developing specific therapeutic strategies. As summarised in Table 1, modulation of either NET production/removal or complement inhibition may represent possible effective strategies in the treatment of autoimmune disease.

### 4.1. Modulation of NET Production

Possible strategies to limit the initial stages of NETosis target reactive oxygen species (ROS) with common treatments, such as N-acetylcysteine and/or NADPH oxidase inhibitors, which have demonstrated modest clinical effects [60]. Colchicine, an anti-inflammatory agent widely used in gout and pericarditis, has also been reported to reduce ROS production and NETosis, thereby limiting neutrophil hyperactivation [61]. Inhibitors of myeloperoxidase, such as 4-aminobenzoic acid hydrazide, have been tested in murine models of SLE and vasculitis complicated by glomerulonephritis, revealing a notable reduction in neutrophil accumulation in glomeruli and coincident proteinuria reduction [34]. An alternative approach to mitigate NET formation is the inhibition of peptidylarginine deiminase 4 (PAD4) [62]. This strategy has been investigated in several animal models, including mice with PAD4 deficiency (PAD4−/−). In PAD4-deficient mice exposed to organ pathologies inducing pulmonary inflammation and acute respiratory distress syndrome, a significant decrease in NETosis, reduced neutrophil infiltration in the lungs, and improved survival were observed compared to wild-type mice [63]. In a different murine SLE model characterized by increased serum levels of interferon-1, treatment with Cl-amidine resulted in reduced NET formation and circulating autoantibodies, with a concomitant normalization of serum complement components. These modifications were followed by diminished IgG deposition within glomeruli, suggesting a potential renoprotective effect [64,65]. In a recent investigation, a biologically active compound library was systematically screened using a high-content imaging assay, leading to the identification of vanilloids as a novel class of NET inhibitors. Vanilloids were found to limit the NET release induced by phorbol myristate acetate and ionomycin while also reducing cytosolic ROS production, underscoring their dual role in limiting oxidative stress and NETosis [66]. Other potential NETosis-modulating targets include actin cytoskeleton regulation, chemokine (C-X-C motif) ligand 5, integrins, and tumor necrosis factor, all of which present promising therapeutic avenues for disorders characterized by dysregulated NET formation [67,68,69].

### 4.2. Modulation of NET Removal

In the last two decades, inhaled recombinant DNase I has been used in patients with cystic fibrosis and other inflammatory pulmonary conditions, demonstrating therapeutic efficacy with minor adverse events [70]. DNase I administration has also been explored for neurodegenerative disorders, such as end-stage Alzheimer’s disease, due to its potential to clear extracellular DNA accumulations [71,72]. In autoimmune diseases, DNase therapy has been explored in murine SLE models, with contrasting results. Recombinant DNase I administration led to a reduction in autoantibody production, amelioration of proteinuria, and decreased kidney damage in a lupus-prone murine model [73]. While patients with SLE tolerated recombinant DNase I administration, further studies are needed to validate its clinical efficacy [74]. Additionally, the presence of anti-DNase antibodies in SLE patients may limit therapeutic efficacy, prompting innovative approaches such as the combination of DNase therapy with anti-DNase antibody blockade to enhance NET clearance and reduce inflammation [75].

### 4.3. Complement Component Inhibitors

In recent years, there has been a surge in pharmaceutical interest in advancing complement inhibitors across diverse indications [76]. This heightened attention is notably underpinned by the exceptional outcomes observed with the anti-C5 blocking antibody eculizumab, which stands as the pioneering complement inhibitor, initially approved for paroxysmal nocturnal hemoglobinuria (PNH) and subsequently for atypical hemolytic uremic syndrome (aHUS) [77]. This anti-C5 humanized monoclonal antibody hinders C5 cleavage by the C5 convertase, thereby impeding the terminal complement effector pathway. Its interference prevents the assembly of the membrane attack complex in the absence of C5b [77].

The European Medicines Agency and the US Food and Drug Administration granted approval for eculizumab in the treatment of aHUS. This endorsement was based on findings from two separate prospective trials involving 17 aHUS patients with thrombocytopenia and 20 aHUS patients needing persistent plasma exchange, respectively [78]. Limited case reports have documented favorable outcomes with eculizumab in managing refractory lupus nephritis [79,80]. In a comprehensive review encompassing SLE patients with renal involvement, irrespective of concurrent lupus nephritis, all six subjects exhibited sustained improvement in renal function and complement parameter normalization post-eculizumab treatment at nine-month follow-up [81]. This favorable response extended to patients with refractory thrombotic microangiopathy linked to SLE [82]. One more randomized phase II trial Eculizumab in patients with ANCA-associated vasculitis is recruiting participants (NCT01275287).

Of note, a multicenter, phase II enrolling study (NCT04564339) is evaluating the efficacy and safety of ravulizumab, a more recent humanized monoclonal antibody that inhibits complement C5, in adults with lupus nephritis. Additionally, an impending clinical trial in the enrollment phase aims to evaluate the effectiveness and safety of LNP023/Iptacopan, a factor B inhibitor, in individuals with active lupus nephritis of Class III–IV, with or without Class V involvement (NCT05268289).

CCX168 (Avacopan), a small molecule C5aR inhibitor, was investigated in a randomized, double-blind, placebo-controlled trial by Jayne et al. [83] The study involved 67 adults with AAV who were treated with Avacopan with or without steroids, alongside cyclophosphamide or the anti-CD20 monoclonal antibody rituximab. Remarkably, treatment responses were observed in 86% and 81% of the Avacopan with and without steroid groups, respectively, while the control group exhibited a 70% response rate, meeting non-inferiority criteria [83]. More recently, same authors reported similar results in randomised clinical trials on 331 patients with AAV: Avacopan proved non-inferior but not superior to prednisone taper in terms of remission at week 26 and was superior to prednisone taper with respect to sustained remission at week 52. All the patients received cyclophosphamide or rituximab [84].

### 4.4. Combined Therapies Modulating Complement Components and NETs

The interplay between complement activation and NETosis has significant implications for therapeutic strategies, particularly in autoimmune and inflammatory diseases such as SLE, AAV, and APS. As widely reported, NETs can amplify complement activation, while complement-derived factors promote NET formation, creating a vicious cycle of inflammation and tissue damage [67]. This bidirectional relationship suggests that simultaneously targeting complement components and NETs may provide synergistic benefits, reducing immune-mediated tissue injury and improving long-term disease outcomes.

Although still in early-stage research, some combination treatment strategies have been explored in preclinical and clinical studies. C5 inhibitors such as Eculizumab and Ravulizumab, in combination with DNase I therapy, have been proposed to reduce complement-driven inflammation while promoting NET degradation, potentially benefiting SLE and APS patients [85]. Similarly, PAD4 inhibitors including Cl-amidine and GSK484, combined with C5a receptor antagonists like Avacopan, have been studied in AAV models, demonstrating reduced NET formation and complement-mediated glomerular injury [86,87]. Despite the therapeutic potential, dual complement–NET targeting carries risks that must be carefully evaluated. Since both NETs and complement components are essential components of innate immunity, their inhibition may lead to increased susceptibility to infections, particularly encapsulated bacterial infections such as *Neisseria meningitidis*, which is a known risk associated with C5 inhibitors [88]. Additionally, since NETs contribute to tissue remodelling and repair, excessive inhibition may result in delayed wound healing or tissue regeneration impairments [89]. Another potential concern is the risk of thrombosis or bleeding complications, as both the complement cascade and NETs play key roles in coagulation homeostasis, and their modulation may shift the balance toward either a pro-thrombotic or hemorrhagic state, particularly when heparin or anticoagulants are involved [67].

In conclusion, the development of dual complement–NET inhibition therapies represents an exciting and promising frontier in the treatment of immune-mediated diseases. However, rigorous clinical trials are required to determine the optimal balance between immune suppression and host defense mechanisms, ensuring therapeutic efficacy limiting adverse effects.

## 5. Conclusions

In the intricate landscape of immune-related diseases, the collaborative dynamics between NETs and complement components play a pivotal role. This synergistic alliance extends beyond localized injury sites to systemic circulation. In autoimmune disorders, such as SLE and AAV, the orchestrated interplay of the complement cascade and NETs can significantly influence disease progression. While the past decade has witnessed the development of targeted drugs focusing on complement components, the modulation of NETosis remains less charted. Exploring and understanding the intricate interconnections between NETs and complement pathways may unveil novel therapeutic avenues for chronic autoimmune conditions.

## Figures and Tables

**Table 1:** **Main Therapeutical Strategies for modulating NETs and Complement.**

Name	Class	Disease	Main Pathogenic Mechanism	Status
***Modulation of NET production***				
N-acetylcysteine	ROS inhibitor	Vasculitis, SLE APS	Reduces oxidative stress-induced NETosis	On the market
PAD4 inhibitors (Cl-amidine, GSK484)	Anti-inflammatory small molecule	Vasculitis, SLE APS	Prevents excessive NET release by neutrophils	Clinical trial Phase I-II
Colchicine, Hydroxychloroquine	Neutrophil inhibitor	SLE	Suppresses neutrophil activation and NET release	On the market
Capsaicin, Eugenol, Vanillin	Phytochemical	Vasculitis, SLE	Reduces NETosis by inhibiting ROS production and neutrophil activation	Pre-clinical studies
***Modulation of NET removal***				
Recombinant DNase I (Dornase alfa)	Enzyme	Vasculitis, SLE APS	Degrades extracellular DNA, promoting NET clearance	Clinical trial Phase I-II
***Complement Inhibitors***				
Eculizumab	Humanized monoclonal antibody	aHUS	Binds C5 to prevent generation of MAC	On the market
Ravalizumab	Humanized monoclonal antibody	aHUS	Binds C5 to prevent generation of MAC	On the market
CCX168 (Avacopan)	Anti-inflammatory small molecule	Vasculitis	Selective inhibitor of the complement C5a receptor	Clinical trial Phase III

aHUS, hemolytic uremic syndrome; APS, antiphospholipid antibody syndrome; ROS, reactive oxygen species; SLE, systemic lupus erythematosus.

## References

[1] Walport M.J. (2001). Complement. First of two parts. N. Engl. J. Med..

[2] Walport M.J. (2001). Complement. Second of two parts. N. Engl. J. Med..

[3] Brinkmann V., Reichard U., Goosmann C., Fauler B., Uhlemann Y., Weiss D.S., Weinrauch Y., Zychlinsky A. (2004). Neutrophil extracellular traps kill bacteria. Science.

[4] Wan Y., Shen J., Ouyang J., Dong P., Hong Y., Liang L., Liu J. (2022). Bibliometric and visual analysis of neutrophil extracellular traps from 2004 to 2022. Front Immunol.

[5] Pinegin B., Vorobjeva N., Pinegin V. (2015). Neutrophil extracellular traps and their role in the development of chronic inflammation and autoimmunity. Autoimmun. Rev..

[6] Vorobjeva N., Dagil Y., Pashenkov M., Pinegin B., Chernyak B. (2023). Protein kinase C isoforms mediate the formation of neutrophil extracellular traps. Int. Immunopharmacol..

[7] Guglietta S., Chiavelli A., Zagato E., Krieg C., Gandini S., Ravenda P.S., Bazolli B., Lu B., Penna G., Rescigno M. (2016). Coagulation induced by C3aR-dependent NETosis drives protumorigenic neutrophils during small intestinal tumorigenesis. Nat. Commun..

[8] Karpati E., Kremlitzka M., Sandor N., Hajnal D., Schneider A.E., Jozsi M. (2021). Complement Factor H Family Proteins Modulate Monocyte and Neutrophil Granulocyte Functions. Front. Immunol..

[9] Schneider A.E., Sandor N., Karpati E., Jozsi M. (2016). Complement factor H modulates the activation of human neutrophil granulocytes and the generation of neutrophil extracellular traps. Mol. Immunol..

[10] Palmer L.J., Damgaard C., Holmstrup P., Nielsen C.H. (2016). Influence of complement on neutrophil extracellular trap release induced by bacteria. J. Periodontal Res..

[11] O’Flynn J., van der Pol P., Dixon K.O., Prohaszka Z., Daha M.R., van Kooten C. (2016). Monomeric C-reactive protein inhibits renal cell-directed complement activation mediated by properdin. Am. J. Physiol. Renal Physiol..

[12] Dixon K.O., O’Flynn J., Klar-Mohamad N., Daha M.R., van Kooten C. (2017). Properdin and factor H production by human dendritic cells modulates their T-cell stimulatory capacity and is regulated by IFN-gamma. Eur. J. Immunol..

[13] O’Flynn J., Dixon K.O., Faber Krol M.C., Daha M.R., van Kooten C. (2014). Myeloperoxidase directs properdin-mediated complement activation. J. Innate Immun..

[14] Vogt W. (1996). Complement activation by myeloperoxidase products released from stimulated human polymorphonuclear leukocytes. Immunobiology.

[15] de Bont C.M., Boelens W.C., Pruijn G.J.M. (2019). NETosis, complement, and coagulation: A triangular relationship. Cell Mol. Immunol..

[16] Maison C.M., Villiers C.L., Colomb M.G. (1991). Proteolysis of C3 on U937 cell plasma membranes. Purification of cathepsin G. J. Immunol..

[17] Wang H., Wang C., Zhao M.H., Chen M. (2015). Neutrophil extracellular traps can activate alternative complement pathways. Clin. Exp. Immunol..

[18] Leffler J., Martin M., Gullstrand B., Tyden H., Lood C., Truedsson L., Bengtsson A.A., Blom A.M. (2012). Neutrophil extracellular traps that are not degraded in systemic lupus erythematosus activate complement exacerbating the disease. J. Immunol..

[19] Yuen J., Pluthero F.G., Douda D.N., Riedl M., Cherry A., Ulanova M., Kahr W.H., Palaniyar N., Licht C. (2016). NETosing Neutrophils Activate Complement Both on Their Own NETs and Bacteria via Alternative and Non-alternative Pathways. Front. Immunol..

[20] Halder L.D., Abdelfatah M.A., Jo E.A., Jacobsen I.D., Westermann M., Beyersdorf N., Lorkowski S., Zipfel P.F., Skerka C. (2016). Factor H Binds to Extracellular DNA Traps Released from Human Blood Monocytes in Response to Candida albicans. Front. Immunol..

[21] Fanouriakis A., Kostopoulou M., Andersen J., Aringer M., Arnaud L., Bae S.C., Boletis J., Bruce I.N., Cervera R., Doria A. (2024). EULAR recommendations for the management of systemic lupus erythematosus: 2023 update. Ann. Rheum. Dis..

[22] Kidney Disease: Improving Global Outcomes (KDIGO) (2021). KDIGO 2021 Clinical Practice Guideline for the Management of Glomerular Diseases. Kidney Int.

[23] Barrat F.J., Crow M.K., Ivashkiv L.B. (2019). Interferon target-gene expression and epigenomic signatures in health and disease. Nat. Immunol..

[24] Herrada A.A., Escobedo N., Iruretagoyena M., Valenzuela R.A., Burgos P.I., Cuitino L., Llanos C. (2019). Innate Immune Cells’ Contribution to Systemic Lupus Erythematosus. Front. Immunol..

[25] Lodi L., Mastrolia M.V., Bello F., Rossi G.M., Angelotti M.L., Crow Y.J., Romagnani P., Vaglio A. (2022). Type I interferon-related kidney disorders. Kidney Int..

[26] Wigerblad G., Kaplan M.J. (2023). Neutrophil extracellular traps in systemic autoimmune and autoinflammatory diseases. Nat. Rev. Immunol..

[27] Alarcon G.S., McGwin G., Petri M., Reveille J.D., Ramsey-Goldman R., Kimberly R.P., PROFILE Study Group (2002). Baseline characteristics of a multiethnic lupus cohort: PROFILE. Lupus.

[28] Tektonidou M.G., Dasgupta A., Ward M.M. (2016). Risk of End-Stage Renal Disease in Patients With Lupus Nephritis, 1971–2015: A Systematic Review and Bayesian Meta-Analysis. Arthritis Rheumatol..

[29] Contreras G., Mattiazzi A., Guerra G., Ortega L.M., Tozman E.C., Li H., Tamariz L., Carvalho C., Kupin W., Ladino M. (2010). Recurrence of lupus nephritis after kidney transplantation. J. Am. Soc. Nephrol..

[30] Bertelli R., Schena F., Antonini F., Reverberi D., Signa S., Pedemonte N., Consolaro A., Gattorno M., Negrini S., Pupo F. (2021). Neutrophil Extracellular Traps in Systemic Lupus Erythematosus Stimulate IgG2 Production From B Lymphocytes. Front. Med..

[31] Nakazawa D., Shida H., Kusunoki Y., Miyoshi A., Nishio S., Tomaru U., Atsumi T., Ishizu A. (2016). The responses of macrophages in interaction with neutrophils that undergo NETosis. J. Autoimmun..

[32] Bruschi M., Petretto A., Bertelli R., Galetti M., Bonanni A., Pratesi F., Migliorini P., Candiano G., Vaglio A., Ghiggeri G.M. (2017). Post-translational modified proteins are biomarkers of autoimmune-processes: NETosis and the inflammatory-autoimmunity connection. Clin. Chim. Acta.

[33] Bruschi M., Petretto A., Santucci L., Vaglio A., Pratesi F., Migliorini P., Bertelli R., Lavarello C., Bartolucci M., Candiano G. (2019). Neutrophil Extracellular Traps protein composition is specific for patients with Lupus nephritis and includes methyl-oxidized alphaenolase (methionine sulfoxide 93). Sci. Rep..

[34] Bruschi M., Moroni G., Sinico R.A., Franceschini F., Fredi M., Vaglio A., Cavagna L., Petretto A., Pratesi F., Migliorini P. (2021). Neutrophil Extracellular Traps in the Autoimmunity Context. Front. Med..

[35] Yu Y., Su K. (2013). Neutrophil Extracellular Traps and Systemic Lupus Erythematosus. J. Clin. Cell Immunol..

[36] Kahlenberg J.M., Carmona-Rivera C., Smith C.K., Kaplan M.J. (2013). Neutrophil extracellular trap-associated protein activation of the NLRP3 inflammasome is enhanced in lupus macrophages. J. Immunol..

[37] Bruschi M., Bonanni A., Petretto A., Vaglio A., Pratesi F., Santucci L., Migliorini P., Bertelli R., Galetti M., Belletti S. (2020). Neutrophil Extracellular Traps Profiles in Patients with Incident Systemic Lupus Erythematosus and Lupus Nephritis. J. Rheumatol..

[38] Wang W., Su J., Yan M., Pan J., Zhang X. (2023). Neutrophil extracellular traps in autoimmune diseases: Analysis of the knowledge map. Front. Immunol..

[39] Takagi H., Arimura K., Uto T., Fukaya T., Nakamura T., Choijookhuu N., Hishikawa Y., Sato K. (2016). Plasmacytoid dendritic cells orchestrate TLR7-mediated innate and adaptive immunity for the initiation of autoimmune inflammation. Sci. Rep..

[40] Demkow U. (2023). Molecular Mechanisms of Neutrophil Extracellular Trap (NETs) Degradation. Int. J. Mol. Sci..

[41] Zuo Y., Yalavarthi S., Gockman K., Madison J.A., Gudjonsson J.E., Kahlenberg J.M., Joseph McCune W., Bockenstedt P.L., Karp D.R., Knight J.S. (2020). Anti-Neutrophil Extracellular Trap Antibodies and Impaired Neutrophil Extracellular Trap Degradation in Antiphospholipid Syndrome. Arthritis Rheumatol..

[42] Reshetnyak T., Nurbaeva K. (2023). The Role of Neutrophil Extracellular Traps (NETs) in the Pathogenesis of Systemic Lupus Erythematosus and Antiphospholipid Syndrome. Int. J. Mol. Sci..

[43] Mathapathi S., Chu C.Q. (2022). Contribution of Impaired DNASE1L3 Activity to Anti-DNA Autoantibody Production in Systemic Lupus Erythematosus. Rheumatol. Immunol. Res..

[44] Almlof J.C., Nystedt S., Leonard D., Eloranta M.L., Grosso G., Sjowall C., Bengtsson A.A., Jonsen A., Gunnarsson I., Svenungsson E. (2019). Whole-genome sequencing identifies complex contributions to genetic risk by variants in genes causing monogenic systemic lupus erythematosus. Hum. Genet..

[45] Yasutomo K., Horiuchi T., Kagami S., Tsukamoto H., Hashimura C., Urushihara M., Kuroda Y. (2001). Mutation of DNASE1 in people with systemic lupus erythematosus. Nat. Genet..

[46] Kisla Ekinci R.M., Balci S., Ozcan D., Atmis B., Bisgin A. (2021). Monogenic lupus due to DNASE1L3 deficiency in a pediatric patient with urticarial rash, hypocomplementemia, pulmonary hemorrhage, and immune-complex glomerulonephritis. Eur. J. Med. Genet..

[47] Hartl J., Serpas L., Wang Y., Rashidfarrokhi A., Perez O.A., Sally B., Sisirak V., Soni C., Khodadadi-Jamayran A., Tsirigos A. (2021). Autoantibody-mediated impairment of DNASE1L3 activity in sporadic systemic lupus erythematosus. J. Exp. Med..

[48] Noubouossie D.F., Whelihan M.F., Yu Y.B., Sparkenbaugh E., Pawlinski R., Monroe D.M., Key N.S. (2017). In vitro activation of coagulation by human neutrophil DNA and histone proteins but not neutrophil extracellular traps. Blood.

[49] Ribon M., Seninet S., Mussard J., Sebbag M., Clavel C., Serre G., Boissier M.C., Semerano L., Decker P. (2019). Neutrophil extracellular traps exert both pro- and anti-inflammatory actions in rheumatoid arthritis that are modulated by C1q and LL-37. J. Autoimmun..

[50] Jarrot P.A., Kaplanski G. (2016). Pathogenesis of ANCA-associated vasculitis: An update. Autoimmun. Rev..

[51] Shiratori-Aso S., Nakazawa D. (2023). The involvement of NETs in ANCA-associated vasculitis. Front. Immunol..

[52] Huang S.U., O’Sullivan K.M. (2022). The Expanding Role of Extracellular Traps in Inflammation and Autoimmunity: The New Players in Casting Dark Webs. Int. J. Mol. Sci..

[53] Kambas K., Chrysanthopoulou A., Vassilopoulos D., Apostolidou E., Skendros P., Girod A., Arelaki S., Froudarakis M., Nakopoulou L., Giatromanolaki A. (2014). Tissue factor expression in neutrophil extracellular traps and neutrophil derived microparticles in antineutrophil cytoplasmic antibody associated vasculitis may promote thromboinflammation and the thrombophilic state associated with the disease. Ann. Rheum. Dis..

[54] Nakazawa D., Tomaru U., Yamamoto C., Jodo S., Ishizu A. (2012). Abundant neutrophil extracellular traps in thrombus of patient with microscopic polyangiitis. Front. Immunol..

[55] Xia Y., He J., Zhang H., Wang H., Tetz G., Maguire C.A., Wang Y., Onuma A., Genkin D., Tetz V. (2020). AAV-mediated gene transfer of DNase I in the liver of mice with colorectal cancer reduces liver metastasis and restores local innate and adaptive immune response. Mol. Oncol..

[56] Patriarcheas V., Tsamos G., Vasdeki D., Kotteas E., Kollias A., Nikas D., Kaiafa G., Dimakakos E. (2025). Antiphospholipid Syndrome: A Comprehensive Clinical Review. J. Clin. Med..

[57] Oku K., Amengual O., Hisada R., Ohmura K., Nakagawa I., Watanabe T., Bohgaki T., Horita T., Yasuda S., Atsumi T. (2016). Autoantibodies against a complement component 1 q subcomponent contribute to complement activation and recurrent thrombosis/pregnancy morbidity in anti-phospholipid syndrome. Rheumatology.

[58] Chen Y., Li X., Lin X., Liang H., Liu X., Zhang X., Zhang Q., Zhou F., Yu C., Lei L. (2022). Complement C5a induces the generation of neutrophil extracellular traps by inhibiting mitochondrial STAT3 to promote the development of arterial thrombosis. Thromb. J..

[59] Knight J.S., Kanthi Y. (2022). Mechanisms of immunothrombosis and vasculopathy in antiphospholipid syndrome. Semin. Immunopathol..

[60] Leung H.H.L., Perdomo J., Ahmadi Z., Yan F., McKenzie S.E., Chong B.H. (2021). Inhibition of NADPH oxidase blocks NETosis and reduces thrombosis in heparin-induced thrombocytopenia. Blood Adv..

[61] Bulnes J.F., Gonzalez L., Velasquez L., Orellana M.P., Venturelli P.M., Martinez G. (2024). Role of inflammation and evidence for the use of colchicine in patients with acute coronary syndrome. Front. Cardiovasc. Med..

[62] Knight J.S., Luo W., O’Dell A.A., Yalavarthi S., Zhao W., Subramanian V., Guo C., Grenn R.C., Thompson P.R., Eitzman D.T. (2014). Peptidylarginine deiminase inhibition reduces vascular damage and modulates innate immune responses in murine models of atherosclerosis. Circ. Res..

[63] Biron B.M., Chung C.S., Chen Y., Wilson Z., Fallon E.A., Reichner J.S., Ayala A. (2018). PAD4 Deficiency Leads to Decreased Organ Dysfunction and Improved Survival in a Dual Insult Model of Hemorrhagic Shock and Sepsis. J. Immunol..

[64] Shen Y., You Q., Wu Y., Wu J. (2022). Inhibition of PAD4-mediated NET formation by cl-amidine prevents diabetes development in nonobese diabetic mice. Eur. J. Pharmacol..

[65] Biron B.M., Chung C.S., O’Brien X.M., Chen Y., Reichner J.S., Ayala A. (2017). Cl-Amidine Prevents Histone 3 Citrullination and Neutrophil Extracellular Trap Formation, and Improves Survival in a Murine Sepsis Model. J. Innate Immun..

[66] Sondo E., Bertelli R., Pesce E., Ghiggeri G.M., Pedemonte N. (2019). High-Content Screening Identifies Vanilloids as a Novel Class of Inhibitors of NET Formation. Front. Immunol..

[67] Wang H., Kim S.J., Lei Y., Wang S., Wang H., Huang H., Zhang H., Tsung A. (2024). Neutrophil extracellular traps in homeostasis and disease. Signal Transduct. Target. Ther..

[68] Fan X., Shu P., Wang Y., Ji N., Zhang D. (2023). Interactions between neutrophils and T-helper 17 cells. Front. Immunol..

[69] Wang S., Song Y., Wang Z., Chang X., Wu H., Yan Z., Wu J., He Z., Kang L., Hu W. (2024). Neutrophil-derived PAD4 induces citrullination of CKMT1 exacerbates mucosal inflammation in inflammatory bowel disease. Cell Mol. Immunol..

[70] Zahm J.M., Debordeaux C., Maurer C., Hubert D., Dusser D., Bonnet N., Lazarus R.A., Puchelle E. (2001). Improved activity of an actin-resistant DNase I variant on the cystic fibrosis airway secretions. Am. J. Respir. Crit. Care Med..

[71] Smalheiser N.R. (2019). Mining Clinical Case Reports to Identify New Lines of Investigation in Alzheimer’s Disease: The Curious Case of DNase I. J. Alzheimers Dis. Rep..

[72] Wallace C.J. (1993). The curious case of protein splicing: Mechanistic insights suggested by protein semisynthesis. Protein Sci..

[73] Manderson A.P., Carlucci F., Lachmann P.J., Lazarus R.A., Festenstein R.J., Cook H.T., Walport M.J., Botto M. (2006). The in vivo expression of actin/salt-resistant hyperactive DNase I inhibits the development of anti-ssDNA and anti-histone autoantibodies in a murine model of systemic lupus erythematosus. Arthritis Res. Ther..

[74] Davis J.C., Manzi S., Yarboro C., Rairie J., McInnes I., Averthelyi D., Sinicropi D., Hale V.G., Balow J., Austin H. (1999). Recombinant human Dnase I (rhDNase) in patients with lupus nephritis. Lupus.

[75] Wang Y., Xiao S., Xia Y., Wang H. (2022). The Therapeutic Strategies for SLE by Targeting Anti-dsDNA Antibodies. Clin. Rev. Allergy Immunol..

[76] Marinho A., Delgado Alves J., Fortuna J., Faria R., Almeida I., Alves G., Araujo Correia J., Campar A., Brandao M., Crespo J. (2023). Biological therapy in systemic lupus erythematosus, antiphospholipid syndrome, and Sjogren’s syndrome: Evidence- and practice-based guidance. Front. Immunol..

[77] Rother R.P., Rollins S.A., Mojcik C.F., Brodsky R.A., Bell L. (2007). Discovery and development of the complement inhibitor eculizumab for the treatment of paroxysmal nocturnal hemoglobinuria. Nat. Biotechnol..

[78] Legendre C.M., Licht C., Muus P., Greenbaum L.A., Babu S., Bedrosian C., Bingham C., Cohen D.J., Delmas Y., Douglas K. (2013). Terminal complement inhibitor eculizumab in atypical hemolytic-uremic syndrome. N. Engl. J. Med..

[79] Pickering M.C., Ismajli M., Condon M.B., McKenna N., Hall A.E., Lightstone L., Terence Cook H., Cairns T.D. (2015). Eculizumab as rescue therapy in severe resistant lupus nephritis. Rheumatology.

[80] Ono M., Ohashi N., Namikawa A., Katahashi N., Ishigaki S., Tsuji N., Isobe S., Iwakura T., Sakao Y., Tsuji T. (2018). A Rare Case of Lupus Nephritis Presenting as Thrombotic Microangiopathy with Diffuse Pseudotubulization Possibly Caused by Atypical Hemolytic Uremic Syndrome. Intern. Med..

[81] Sciascia S., Radin M., Yazdany J., Tektonidou M., Cecchi I., Roccatello D., Dall’Era M. (2017). Expanding the therapeutic options for renal involvement in lupus: Eculizumab, available evidence. Rheumatol. Int..

[82] Wright R.D., Bannerman F., Beresford M.W., Oni L. (2020). A systematic review of the role of eculizumab in systemic lupus erythematosus-associated thrombotic microangiopathy. BMC Nephrol..

[83] Jayne D.R.W., Bruchfeld A.N., Harper L., Schaier M., Venning M.C., Hamilton P., Burst V., Grundmann F., Jadoul M., Szombati I. (2017). Randomized Trial of C5a Receptor Inhibitor Avacopan in ANCA-Associated Vasculitis. J. Am. Soc. Nephrol..

[84] Berdunov V., Ramirez de Arellano A., Li T., Vintderdag H., Baxter G. (2024). Key considerations for modelling the long-term costs and benefits of treatments for ANCA-associated vasculitis. Clin. Exp. Rheumatol..

[85] Risitano A.M., Peffault de Latour R., Marano L., Frieri C. (2022). Discovering C3 targeting therapies for paroxysmal nocturnal hemoglobinuria: Achievements and pitfalls. Semin. Immunol..

[86] D’Alessandro M., Conticini E., Bergantini L., Cameli P., Cantarini L., Frediani B., Bargagli E. (2022). Neutrophil Extracellular Traps in ANCA-Associated Vasculitis and Interstitial Lung Disease: A Scoping Review. Life.

[87] O’Sullivan K.M., Holdsworth S.R. (2021). Neutrophil Extracellular Traps: A Potential Therapeutic Target in MPO-ANCA Associated Vasculitis?. Front. Immunol..

[88] Herrmann J.B., Muenstermann M., Strobel L., Schubert-Unkmeir A., Woodruff T.M., Gray-Owen S.D., Klos A., Johswich K.O. (2018). Complement C5a Receptor 1 Exacerbates the Pathophysiology of *N. meningitidis Sepsis* and Is a Potential Target for Disease Treatment. mBio.

[89] Zhu S., Yu Y., Ren Y., Xu L., Wang H., Ling X., Jin L., Hu Y., Zhang H., Miao C. (2021). The emerging roles of neutrophil extracellular traps in wound healing. Cell Death Dis..

